# Decision time modulates social foraging success in wild common ravens, *Corvus corax*

**DOI:** 10.1111/eth.12986

**Published:** 2019-11-24

**Authors:** Mario Gallego‐Abenza, Matthias‐Claudio Loretto, Thomas Bugnyar

**Affiliations:** ^1^ Department of Cognitive Biology University of Vienna Vienna Austria; ^2^ Konrad Lorenz Forschungsstelle Core Facility for Behaviour and Cognition University of Vienna Grünau im Almtal Austria; ^3^ Department of Migration Max Planck Institute of Animal Behavior Konstanz Germany; ^4^ Department of Biology University of Konstanz Konstanz Germany

**Keywords:** cognition, *Corvus corax*, decision‐making, kleptoparasitism, scrounging, social foraging

## Abstract

Social foraging provides several benefits for individuals but also bears the potential costs of higher competition. In some species, such competition arises through kleptoparasitism, that is when an animal takes food which was caught or collected by a member of its social group. Except in the context of caching, few studies have investigated how individuals avoid kleptoparasitism, which could be based on physical strength/dominance but also cognitive skills. Here, we investigated the foraging success of wild common ravens, *Corvus corax*, experiencing high levels of kleptoparasitism from conspecifics when snatching food from the daily feedings of captive wild boars in a game park in the Austrian Alps. Success in keeping the food depended mainly on the individuals’ age class and was positively correlated with the time to make a decision in whether to fly off with food or consume it on site. While the effect of age class suggests that dominant and/or experienced individuals are better in avoiding kleptoparasitism, the effect of decision time indicates that individuals benefit from applying cognition to such decision‐making, independently of age class. We discuss our findings in the context of the ecological and social intelligence hypotheses referring to the development of cognitive abilities. We conclude that investigating which factors underline kleptoparasitism avoidance is a promising scenario to test specific predictions derived from these hypotheses.

## 1 INTRODUCTION

During foraging, group formation has many advantages for the individuals: conspecifics and/or heterospecifics may provide information regarding food sources and feeding opportunities (Powell, 1974) or their presence may reduce predation risk (Beauchamp, 2004). However, individuals engaging in group foraging might also experience costs, especially in social species, where group members might represent a source of permanent competition during foraging (Barta & Giraldeau, 1998). This fact leads to potential decline of individual foraging efficiency, especially when food resource becomes limited or group size increases (Goss‐Custard & Durell, 1988). To remain efficient, socially foraging individuals typically adjust their behaviour to those of others (Giraldeau & Caraco, 2000). For instance, in starlings *Sturnus vulgaris*, individuals flexibly use behavioural cues provided by conspecifics for patch assessment, depending on the type of environment in which their foraging takes place (Templeton & Giraldeau, 1995, 1996).

Behavioural plasticity during social foraging is particularly evident when individuals switch between producing food themselves and exploiting the food made available by others (Giraldeau & Caraco, 2000). The latter tactic is termed scrounging (Vickery et al., 1991). Depending on the species’ foraging ecology, scrounging can take different forms (Giraldeau & Caraco, 2000), from exploiting a food patch found by others to directly stealing food from others. Hereby, kleptoparasitism commonly refers to those cases where individuals use force, or threat of force, to obtain the food from others (Baglione & Canestrari, 2009), whereas pilfering refers to those cases where food is stolen out of another individual's cache and physical interactions between cache owner and thief are avoided (Emery & Clayton, 2001; but see Giraldeau & Caraco, 2000 for a slightly different terminology). Game theoretical models have been successfully used to understand the conditions under which individuals adopt producer or scrounger roles (Afshar & Giraldeau, 2014; Giraldeau & Caraco, 2000). Comparative analyses of a large set of field reports in birds indicate that interspecific kleptoparasitism is associated more closely with cognition than with physical power and aggression: the probability that kleptoparasitism is present in an avian family is positively associated with residual brain size but not with body size; likewise, kleptoparasitic species have larger brains than their hosts (Morand‐Ferron, Sol, & Lefebvre, 2007). On the species level, these findings are supported by behavioural observations that explore the cognitive mechanisms underlying scrounging and the countermeasures taken against being exploited by others. In some primates species, low‐ranking individuals tend to reach feeding sites before high‐ranking individuals. This “early arrival” tactic has been described in capuchin monkeys, *Cebu apella* (Di Bitetti & Janson, 2001), Japanese macaques, *Macaca fuscata* (Belisle & Chapais, 2001) and long‐tailed macaques, *Macaca fascicularis* (Dubuc & Chapais, 2007) and is interpreted as subordinates applying social knowledge to avoid competition and/or to increase their foraging efficiency. Furthermore, cases of tactical deception, like withholding information and providing false information (Byrne & Whiten, 1988a), have been reported predominantly in the context of (avoiding) kleptoparasitism. In primates (Byrne & Whiten, 1988b; Coussi‐Korbel, 1994; Hirata & Matsuzawa, 2001; Menzel, 1974), pigs (Held, Mendl, Devereux, & Byrne, 2002) and birds (Bugnyar & Kotrschal, 2004; Flower, Gribble, & Ridley, 2014; Munn, 1986), some individuals (try to) lead conspecifics away from food and/or “cry wolf” in absence of any predator to gain access to food found by others. Such tactics for outwitting competitors are assumed to be cognitively demanding and in line with some of the core hypotheses concerning brain evolution (Byrne, 1997; Byrne & Whiten, 1988b; Dunbar, 2003). Likewise, the interplay between pilfering and avoidance of cache theft, which has been studied particularly in corvids (Clayton, Dally, & Emery, 2007; Heinrich & Pepper, 1998), can be viewed as a producer‐scrounger scenario and has been suggested as one of the driving forces for the advanced socio‐cognitive skills of some of these birds (Bugnyar & Kotrschal, 2002a).

Common ravens *Corvus corax* are scavengers, which form temporary foraging groups at food bonanzas such as carcasses or kills (Heinrich, 1988) as well as at garbage dumps and game parks (Loretto, Schuster, & Bugnyar, 2016); accordingly, the size and composition of foraging groups vary across days (Braun, Walsdorff, Fraser, & Bugnyar, 2012; Heinrich, Kaye, Knight, & Schaumburg, 1994). However, ravens also show substantial individual variation in their local preferences and fission‐fusion dynamics, respectively, with some birds encountering each other regularly (over up to several years) at the foraging site or repeatedly at different sites (Loretto et al., 2017). Furthermore, ravens tend to form affiliative social relationships already at the non‐breeder state, which resemble primate social bonds (Fraser, Schino, & Aureli, 2008) and function as alliances in conflicts (Braun & Bugnyar, 2012; Szipl, Ringler, Spreafico, & Bugnyar, 2017). All these facts indicate that raven foraging groups are not just aggregations but, at least in part, structured by individual spatial preferences and social relationships.

The foraging behaviour of ravens is highly plastic: individuals may actively attract others via calls to food sources that are difficult to access (Bugnyar & Kotrschal, 2001; Heinrich, 1988), which constitutes a form of cooperation on a mutualistic basis where signallers might benefit from enlarging the foraging group and neutralize thus the defence of dominance individuals (Heinrich & Marzluff, 1991). Recruitment can also arise through communal roosting, which serves as information centres for previously encountered food sources (Marzluff, Heinrich, & Marzluff, 1996; Wright, Stone, & Brown, 2003). Aside from these cases of active recruitment, individuals may specialize in exploiting the discoveries of others (Dall & Wright, 2009), opportunistically steal the food acquired from others (Bugnyar & Kotrschal, 2002b) or pilfer the caches made by others (Bugnyar & Kotrschal, 2002a). Raven foraging behaviour can thus be described as producer‐scrounger interactions at different phases during foraging, that is when they search for food, when they try to keep food and when they cache food. Physical strength and dominance status may bias individuals in their choices of tactics, specifically in respect to engaging in kleptoparasitism. Note that raven dominance rank depends not only on individual strength but also on age class (adults >juveniles), sex (males >females) and bonding status (bonded >non‐bonded; Braun & Bugnyar, 2012). Moreover, due to the frequent changes in group composition, the same birds may be dominant in one foraging situation but not in the other.

Social knowledge and experience may help ravens to negotiate such a dynamic social environment, that is deciding when to exploit others and when to avoid being exploited. Furthermore, cognitive skills, such as decision‐making or inhibition control, could allow individuals that are preferred targets of kleptoparasitism to develop countermeasures.

Studies on captive ravens indicate advanced cognition in competition for cached food. While young ravens possess an “innate” motivation to store food for later consumption (Gwinner, 1965), they have to learn when and where they place the caches in order to keep them safe from pilfering (Bugnyar & Kotrschal, 2002a; Bugnyar, Stöwe, & Heinrich, 2007), whereby they may come to comprehend the others’ visual perspective (Bugnyar, 2011; Bugnyar, Reber, & Buckner, 2016). Moreover, ravens have to learn to control their impulse to pilfer others’ caches while the cache owners, or potential competitors, are still present (Bugnyar & Heinrich, 2005, 2006; Bugnyar, Schwab, Schloegl, Kotrschal, & Heinrich, 2007). Aside from caching, ravens have been demonstrated to control their impulsivity in an exchange paradigm, that is instead of consuming an initial food item they later exchange it for one of better quality (Dufour, Wascher, Braun, Miller, & Bugnyar, 2012; Hillemann, Bugnyar, Kotrschal, & Wascher, 2014). Therefore, we could expect prolonged decision time to have some benefits associated with foraging efficiency in wild social birds, in particular in those species with high levels of intra‐specific kleptoparasitism.

In the present study, we focused on the foraging success of individually marked ravens snatching food during zoo animal feedings (i.e., applying a producer tactic). We were interested in the factors determining the individuals’ foraging success (measured as foraging success rate) based on their capability to elude kleptoparasitism from surrounding conspecifics. We hypothesized that raven foraging success would primarily depend on factors associated with dominance status like age class, sex and winning probability in agonistic interactions. Furthermore, we hypothesized that subordinate ravens would suffer from kleptoparasitism and would, therefore, benefit from exhibiting high levels of behavioural plasticity. The efficiency of subordinate individuals should, in this case, be largely determined by the individuals’ experience and cognitive abilities. We thus predicted that the birds’ foraging success rate should not only be age class and sex biased, with older ravens being more efficient than younger and males being more efficient that females, but modulated by behavioural plasticity as a result of learning, decision‐making and impulsivity control. Specifically, and due to known low survival rate of juveniles (in the first year) compared to subadults (2–3 years old; Webb, 2004), we predicted that subadult ravens should be better in coping with kleptoparasitism than juvenile ravens in terms of judging when it is safe to consume food directly on site or when it is better to carry food off. Given the substantial variation in fission‐fusion dynamics in our population, we also expected individuals that spend long periods in the study area (“residents”) to show high foraging efficiency, as they should have a better knowledge of the local social environment than ravens that spend only little time in the study area (“vagrants”).

## 2 MATERIAL AND METHODS

### 2.1 Study species and site

The study took place at Cumberland Wildpark, in Grünau im Almtal, Austria, where common ravens forage at the enclosures of zoo animals all year round in groups of 20–80 birds. These foraging groups are not stable units: while some individuals use the park regularly over several months and even years, others just visit from time to time (Braun et al., 2012; Loretto et al., 2017). The presence and social interactions of these ravens are monitored on a daily basis as part of a long‐term programme. For individual identification, birds are caught in drop‐in traps and marked with rings and patagial wing tags (Caffrey, 2000). During the marking process, age class is determined based on feather colouration and inner beak colour; birds are then categorized as juveniles (<1 year old), subadults (1–3 years old) and adults (>3 years old; Heinrich & Marzluff, 1992). Sex is determined via genetic markers from blood samples.

Over the course of the current study, 46 marked ravens used the zoo as a food source; they represented around 50% of the individually identified ravens present at our study site on a daily basis at that time. For data collection, we chose the enclosure of wild boars *Sus scrofa* as the enclosure's landscape allowed an excellent view of the feeding site and its surroundings. Furthermore, compared to the feedings of large predators like wolves *Canis lupus* and bears *Ursos arctos*, wild boars do not show any aggressive food defence towards ravens (Bugnyar & Kotrschal, 2001; Nácarová, Veselý, & Bugnyar, 2018).

### 2.2 Ethical note

Trapping, blood sampling and marking have been carried out under the licence for animal experimentation of the Austrian government (BMWF‐66.006/0009‐II/3b/2012 and BMBWF‐66.006/0015‐V/3b/2018). As the study itself was non‐invasive and based on behavioural observations only, it was not classified as animal experiment in accordance with the Austrian law (§2. Federal Law Gazette No. 114/ 2012). The monitoring and ringing programme of the Konrad Lorenz Forschungsstelle is authorized by the Central Administration of Upper Austria.

### 2.3 Data collection and analysis

The study was conducted between March 2017 and March 2018. In this one‐year period, we video recorded 143 feedings of wild boars using an action camera (GoPro HD Hero 2 and GoPro HD Hero 5, attached at the fence of the enclosure, 1.5 m height above the food). From these videos, we reported 779 food retention attempts from 46 marked individuals (mean = 16.9 attempts per individual, range = 1–95). Individual food retention attempts concerned two behavioural tactics: carrying food items away from the feeding site or consuming them directly at the feeding site. In either case, we focused on food items larger than a raven beak's length, since small pieces carried inside the beak or throat pouch are not likely to be kleptoparasitized. Kleptoparasitic attacks typically result in some food transfer; however, and due to the difficulty of quantifying on the video how much food each of the ravens got during/after harassment, we thus defined success in food retention when ravens managed to carry a food item away or to consume it in front of any conspecific without being chased or harassed, respectively. A recent observational study focussed on caching locations out of our camera's view, reveals that kleptoparasitism hardly occurs and ravens ensure their caches for later consumption (Beck et al.,2019). For each attempt, we measured the time (in seconds) between grabbing a piece of food and a subsequent decision of either flying off with it or consuming it on site. Further, we measured the distance (in multiples of body length) to the nearest conspecific when grabbing a piece of food and the number of surrounding conspecifics.

In addition to the foraging information scored from the video, we recorded which of the marked individuals were present at our study site every day. Independently to kleptoparasitism events, individual involvement in agonistic interactions was recorded on an *ab libitum* basis via direct observation together with those occurred within the camera's view. We reported a total of 575 dyadic agonistic interactions (mean of agonistic interactions per individual = 12.5, range 3–74), in which the “winner” and “loser” were identified by observing how each dyadic agonistic interaction resolved (see Braun & Bugnyar, 2012 for detailed description of agonistic categories). Inter‐observer reliability was established by coding agonistic interactions from videos with Dr. Szipl, G. as second observer, and reliability was excellent (ICC between 0.997 and 1.0). Since raven non‐breeder groups are characterized by high levels of fission‐fusion dynamics, not all individuals involved in conflicts were individually marked; we thus calculated the “winning probability” for each marked bird by dividing the number of won interactions by the total number of agonistic interactions being involved and used it as a proxy for dominance rank. A previous study conducted in the same study area showed stability of the dominance measures when looking at repeated interactions of individually marked ravens within the same sex, age class and bonding category over 2 years (Braun & Bugnyar, 2012). To obtain a standardized parameter for the individuals’ presence at the study site, we calculated the “percentage of days being present at study site,” a day‐specific value for each individual per feeding protocol based on the percentage of days being present at the study area during the 25 days before and 5 days following the day when the feeding attempts were reported.

### 2.4 Statistical analysis

We used R software (R Core Development Team, 2014) to run our statistical analyses. We performed generalized linear mixed models using “glmmADMB” package (Fournier et al., 2012), including success of keeping the food in both foraging tactics, “overall foraging success,” as response variable (binomial distribution error) and both individual and day as random effects, thus controlling for potential individual differences in being targeted by kleptoparasites (see Table S5). Similarly, we conducted the same modelling approach within each foraging tactic, either flying away with food or consuming it on site. In addition, in order to investigate raven decisions to fly away with food, we included flying away as response variable (binomial distribution error). Number of ravens, distance to nearest conspecific, decision time (s), percentage of days being present at study site, winning probability, age class and sex were included as fixed factors in all models. We z‐transformed all the continuous predictor variables in the full model. We followed an information‐theoretical approach for model selection using “MuMIn package” (Barton, 2019) by calculating all possible models and selecting the best models within ΔAICc ≤ 6 with respect to the top‐ranked model (Burnham & Anderson, 2002; Symonds & Moussalli, 2011). We averaged these models and obtained model‐averaged coefficients following Burnham and Anderson (2002). We used R‐package “car” (Fox & Weisberg, 2011) to test for collinearity of fixed factors before they were entered in the full model, with a resulting variance inflation factor <4 for all variables. We discuss the results based on both effect size and relative importance value (0–1) of each predictor, whereby one refers to the highest contribution in explaining the response variable.

## 3 RESULTS

### 3.1 Descriptive findings

We recorded a total of 779 individual foraging events that were characterized as food retention attempts, that is either carrying food away or eating it on site: out of those, 272 were made by adults (mean of food retention attempts per individual = 22.6, range = 4–95), 366 by subadults (mean of food retention attempts per individual = 17.4, range = 1–91) and 141 by juveniles (mean of food retention attempts per individual = 5.6, range = 1–27). Adults typically attempted to carry food off the site in 241 cases, 89%; subadults attempted on 266 cases, 73%; and juveniles in 70 cases, 50%, Table 1. The two tactics differed in the likelihood of being kleptoparasitized: attempts to carry food off the site received less kleptoparasitism (19%–40%, depending on age class) than attempts to feed on site (71%–86%, Table 1). Regarding sex‐specific differences in kleptoparasitism occurrence, we found similar values in both foraging tactics (24% in males vs. 26% in females when carrying off food; 74% in males vs. 87% in females when feeding on site, Table 1).

**Table 1:** Summary of food retention attempts by marked ravens

Age class	Food retention attempts	Carrying food away attempts	Kleptoparasitized carrying food away attempts (%)	Consuming food on site attempts	Kleptoparasitized consuming food on site attempts (%)
Adult (12 ind.)	272	241	46 (**19.09**)	31	22 (**70.97)**
Subadult (17 ind.)	366	266	75 (**28.19**)	100	80 (**80**)
Juvenile (17 ind.)	141	70	28 (**40**)	71	61 (**85.91**)
Sex					
Male (22 ind.)	283	185	45 (**24.32**)	98	73 (**74.49**)
Female (24 ind.)	496	393	104 (**26.46**)	103	90 (**87.38**)

Note

The table shows the percentage of kleptoparasitism occurring for in each foraging tactic (either carrying food away or consuming food on site) by age class and sex.

### 3.2 Overall foraging success

**Figure 1:**
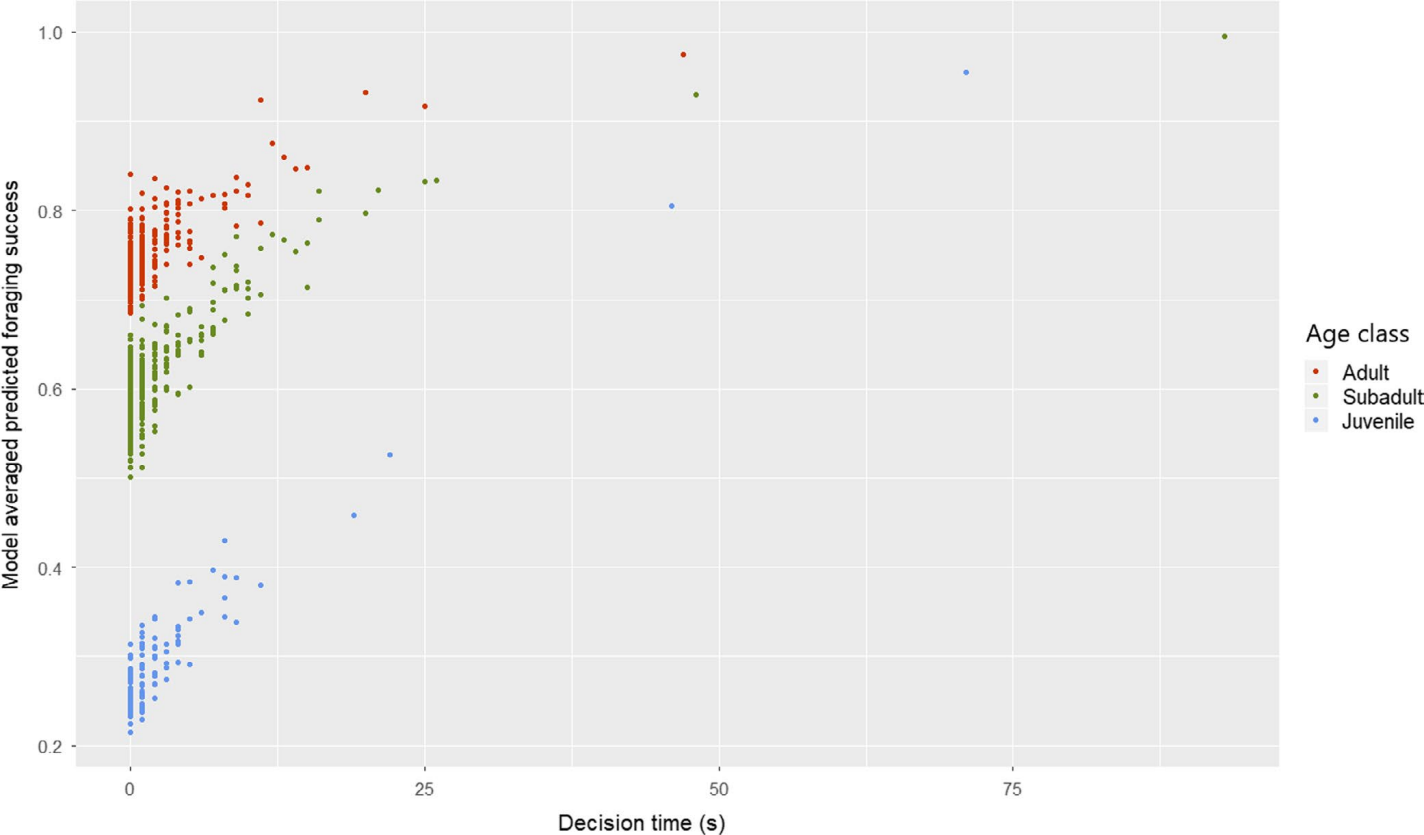
Scatterplot of model‐averaged predicted foraging success, against the decision time (seconds) coloured by age class. Predicted foraging success positively correlates with decision time in all age classes [Colour figure can be viewed at http://www.wileyonlinelibrary.com]

Both age class and decision time had the highest relative importance explaining foraging success in respect to avoiding kleptoparasitism from surrounding conspecifics (see Table 2 for model coefficients). Subadult and juvenile ravens showed lower foraging success than adults. Regarding the decision time, the time ravens took to make a decision (whether to fly off with food or consume it directly on site) had a positive effect on their foraging success (see Figure 1). The distance to the nearest conspecific when grabbing a food item had a slight positive effect on their foraging success. Sex (male) and percentage of days being present at the study site also had a positive effect on the general foraging success of ravens but their relative importance and effect size were negligible low. As expected, the number of surrounding conspecifics at the moment when food retention was attempted had a negative effect on the foraging success; however, it shows a low effect size. Estimates of the full model before model averaging are available in table S1 of Supplementary material.

**Table 2:** Table showing the model‐averaged coefficients

	Estimate	Adjusted SE	CI lower limit (2.5%)	CI upper limit (97.5%)	Relative importance
**Overall foraging success**					
Intercept	1.14	0.29	0.55	1.72	
Age class (Subadult)	−0.72	0.35	−1.41	−0.03	1
Age class (Juvenile)	−2.09	0.44	−2.96	−1.22	1
Distance to conspecific	0.10	0.12	−0.03	0.38	0.59
Decision time (sec)	0.32	0.13	0.07	0.57	1
Number of surrounding ravens	−0.08	0.10	−0.35	0.05	0.52
Sex (male)	0.13	0.27	−0.31	1.02	0.38
Percentage of days being present (0–1)	0.04	0.092	−0.13	0.36	0.36
Winning probability (0–1)	−0.02	0.10	−0.43	0.28	0.28

Note

It shows the coefficients with adjusted standard errors, lower are upper confidence intervals and relative importance values of each fixed factor when modelling the overall foraging success. Factors with a relative importance above 0.6 appear shaded.

### 3.3 Consuming food directly on site

When focusing on those cases in which ravens decided to consume the food directly at the feeding site (Table 3), their success of keeping the food was affected mainly by their distance to nearby conspecifics at the time they took the piece of food and the time they took to make a decision (i.e., to stay rather than fly off). In both cases, these parameters were positively correlated with foraging efficiency. Moreover, social parameters like “winning probability” and “presence” (duration of stay at the study site) became slightly more relevant when consuming food in front of conspecifics. Sex and age class had an effect on foraging success (with old and male individuals being less harassed than juveniles and females) but their relative importance was negligible low. The number of surrounding conspecifics did not affect foraging success when consuming food on site.Estimates of full model before model averaging are available in table S2 of Supplementary material.

**Table 3:** Table showing the model‐averaged coefficients

	Estimate	Adjusted SE	CI lower limit (2.5%)	CI upper limit (97.5%)	Relative importance
**Consuming food on site success**					
Intercept	−13.28	13.11	−38.97	12.41	
Age class (Subadult)	0.66	4.84	−12.84	15.99	0.42
Age class (Juvenile)	−2.81	6.59	−23.89	10.59	0.42
Distance to conspecific	1.49	1.83	−1.27	5.86	0.65
Decision time (sec)	2.50	2.05	−1.50	6.52	1.00
Number of surrounding ravens	0.003	0.09	−0.62	0.69	0.08
Sex (male)	0.59	0.98	−0.31	3.57	0.36
Percentage of days being present (0–1)	0.81	1.59	−2.16	5.42	0.50
Winning probability (0–1)	0.38	0.60	−0.30	2.11	0.42

Note

It shows the coefficients with adjusted standard errors, lower are upper confidence intervals and relative importance values of each fixed factor when modelling the foraging success in consuming food on site. Factors with a relative importance above 0.6 appear shaded.

### 3.4 Decision to carry food away

The ravens’ decision to carry food off the feeding site was positively correlated with the number of surrounding conspecifics (i.e., potential competitors on site; Table 4a). Furthermore, there was a strong effect of age classes: adults and subadults were more likely to carry food away than were juveniles. Apart from these, other fixed factors appeared to not affect relevantly ravens’ decision of carrying off food. Estimates of full model before model averaging are available in table S3 of Supplementary material.

**Table 4:** Summary of model‐averaged coefficients

	Estimate	Adjusted SE	CI lower limit (2.5%)	CI upper limit (97.5%)	Relative importance
**Decision to carry food away**					
Intercept	2.23	0.35	1.54	2.93	
Age class (Subadult)	−0.87	0.41	−1.67	−0.07	1
Age class (Juvenile)	−2.44	0.46	−3.35	−1.53	1
Distance to conspecific	−0.01	0.06	−0.26	0.16	0.27
Decision time (sec)	0.02	0.06	−0.11	0.24	0.30
Number of surrounding ravens	0.28	0.12	0.07	0.51	0.99
Sex (male)	−0.10	0.26	−1.03	0.40	0.32
Percentage of days being present (0–1)	0.0002	0.07	−0.27	0.27	0.25
Winning probability (0–1)	0.01	0.10	−0.34	0.43	0.25
**Success at carrying food away**					
Intercept	1.55	0.24	1.08	2.02	
Age class (Subadult)	−0.50	0.31	−1.11	0.03	0.93
Age class (Juvenile)	−1.06	0.52	−2.01	−0.27	0.93
Distance to conspecific	0.23	0.18	0.001	0.59	0.77
Decision time (sec)	0.08	0.14	−0.13	0.49	0.45
Number of surrounding ravens	−0.34	0.14	−0.59	−0.09	0.98
Sex (male)	0.24	0.29	−0.10	0.95	0.57
Percentage of days being present (0–1)	0.03	0.09	−0.17	0.37	0.31
Winning probability (0–1)	−0.05	0.12	−0.47	0.21	0.35

Note

The table shows the coefficients with adjusted standard errors, lower and upper confidence intervals and relative importance values of each fixed factor when modelling a) the ravens’ decision to carry off food and b) their foraging success when carrying it.

### 3.5 Success at carrying food away

Success in flying off with food meant that ravens carrying food managed to avoid being chased by other conspecifics (Table 4b). The number of surrounding conspecifics had a negative effect on success, as ravens with food were more likely chased when there was a large number of surrounding conspecifics. There was also a strong effect of age class, with adults and subadults receiving fewer chases than did juveniles. The distance to the nearest neighbour (at the time when a focal raven grabbed the food) was positively correlated with success. Here, we found little effect of decision time, presence at the study area or winning probability, each of which had both low effect sizes and low relative importance coefficients. However, sex had a clear effect, with males receiving fewer chases than did females. Estimates of full model before model averaging are available in table S4 of Supplementary material. 

## 4 DISCUSSION

Ravens faced high levels of conspecific kleptoparasitism when snatching food pieces from the feedings of captive wild boars. The success of keeping food depended mainly on the birds’ age class and the amount of time they took to decide whether to fly off with food or consume it directly on site (Figure 1). When modelling the two tactics (flying off and consuming food on site) separately, we found that adults and subadults had an advantage over juveniles when carrying food away, that is they were less likely chased by others. However, age class did not have such an effect on consumption attempts on site; here, the time taken to make a decision (to stay rather than fly off) and the timing of grabbing a food piece (measured as distance to nearest conspecific) were the best predictors of keeping food safe from scroungers.

We predicted that age class would strongly affect foraging success, as adult ravens are known for their high resource holding potential and dominance status in comparison with younger ravens (Heinrich, 1989; Marzluff & Heinrich, 1991). Yet, other factors related to dominance, like the birds’ sex and winning probability in conflicts (Braun & Bugnyar, 2012), had little effect on their success of avoiding kleptoparasitism. Hence, older ravens likely benefited from a combination of both physical strength and experience, particularly when flying off with food. This fits with the age‐specific foraging proficiency shown in most of the avian species (Wunderle, 1991).

Aside from age class, the individual attempts to fly off with food were positively correlated with the number of conspecifics around. Note that ravens typically gathered at the wild boar enclosure already before feeding started, reaching their maximum group size at the beginning of the feeding. We may thus interpret the above‐mentioned correlation directionally, that is that birds tried to leave with food more often when the foraging group was larger. However, the individual success in keeping the food when flying off correlated negatively with the number of conspecifics around, suggesting that the birds had difficulties in escaping kleptoparasitism when the group was large. Hence, carrying food away without being kleptoparasitized represented a challenge for ravens, particularly when they were young and when many conspecifics were around. Ultimately, flying off with food seems to pay off for group foraging ravens (lower kleptoparasitism occurrence, see Table 1), as it allows them to cache food out of sight of potential competitors (Heinrich & Pepper, 1998) and, despite additional costs in time and energy, to secure several loads of food for later consumption (Heinrich, 1988). However, whether all observed food trips resulted in successful food caching and later consumption remains unknown. A similar effect of group size was experimentally shown on coho salmon, *Oncorhynchus kisutch*, whereby, as group size increased, juvenile salmon captured more prey items and ventured closer to the feeder, indicating changes in foraging behaviour driven by group size variation (Grand & Dill, 1999).

Raven success in saving food for immediate consumption did not depend on factors related to dominance or group size but on parameters indicative for cognitive processing, that is the distance to the nearest conspecific when grabbing a piece of food and the time between grabbing a piece of food and making a decision (fly off or consume the food on site). Both parameters correlated positively with success in keeping food, suggesting that the better individuals timed their approach and the longer they waited to decide whether or not to fly off with food, the better they were in avoiding kleptoparasitism on site. In some primate species, flexible timing in feeding has been shown to affect foraging efficiency positively, that is subordinate macaques tend to arrive at the feeding site before than higher‐ranked individuals, this is known as early arrival tactic (*Macaca fuscata*: Belisle & Chapais, 2001; *Macaca fascicularis*: Dubuc & Chapais, 2007). Possibly, ravens used their decision time to assess the current situation of competition, that is the amount of aggression and kleptoparasitism in the immediate surrounding. However, what we measured as “decision time” could also reflect the ravens’ ability to control their impulse to fly off with food. Thus, our findings may support a new avenue for impulse control in ravens shaped by a competitive social foraging scenario. Further studies are needed to distinguish between these alternatives.

Given the substantial spatio‐temporal dynamics in our non‐breeder population, we also expected individuals that spend long periods in the study area (“residents”) to show high foraging success, as they should have a better knowledge of the local social environment than ravens that spend only little time in the study area (“vagrants”). However, our results hardly support this prediction as we found only a weak positive effect of individuals’ presence at the study area on their success of consuming food on site (Table 3). A possible explanation for these results is that ravens face similar social challenges at different foraging sites across their home range. GPS‐tracking revealed that ravens of our study population in the Austrian Alps make heavy use of anthropogenic food sources, that is feedings of game and farm animals, garbage dumps and composting plants (Loretto et al., 2016). At several of those places, they form large groups and potentially face similar levels of competition as at our study site. Avoiding kleptoparasitism would thus be an important skill in their daily life, irrespective of where they forage.

Taken together, our findings support the prediction that foraging ravens show high plasticity in their behaviour. Individuals frequently engaged in producer‐scrounger interactions, whereby individuals in possession of food (producers) became the target of kleptoparasitism by conspecifics (scroungers). Beyond the scope of the producer‐scrounger scenario (already described by Bugnyar & Kotrschal, 2002b), our findings shed light on kleptoparasitism avoidance from the producer's perspective, whereby success in keeping the food seemed to depend on the individuals’ physical abilities and experience (as indicated by the effect of age class) as well as cognitive skills (as indicated by the effect of decision time). These findings are in line with the “foraging cognition hypothesis” (Byrne, 1997; Parker & Gibson, 1977; Rosati, 2017), which emphasizes the need of food acquisition as one of the main driving forces behind the evolution of cognition. However, the findings also fit the “social intelligence hypothesis” (Humphrey, 1976; Jolly, 1966), as interactions with conspecifics seem to be key for shaping the cognitive abilities employed during social foraging. Further research on the foraging skills of common ravens should test predictions derived from both hypotheses, whereby group size or composition and food accessibility can be experimentally modified. Furthermore, longitudinal studies should investigate the development of behavioural tactics to avoid kleptoparasitism and the cognitive skills identified in this study that presumably underlie these behaviours.

## CONFLICT OF INTEREST

None.

## References

[eth12986-bib-0001] Afshar, M. , & Giraldeau, L.‐A. (2014). A unified modelling approach for producer–scrounger games in complex ecological conditions. Animal Behaviour, 96, 167–176. 10.1016/j.anbehav.2014.07.022

[eth12986-bib-0002] Baglione, V. , & Canestrari, D. (2009). Kleptoparasitism and temporal segregation of sympatric corvids foraging in a refuse dump. The Auk, 126(3), 566–578. 10.1525/auk.2009.08146

[eth12986-bib-0003] Barta, Z. , & Giraldeau, L. A. (1998). The effect of dominance hierarchy on the use of alternative foraging tactics: A phenotype‐limited producing‐scrounging game. Behavioral Ecology and Sociobiology, 42(3), 217–223. 10.1007/s002650050433

[eth12986-bib-0004] Barton, K. (2019). MuMIn: Multi‐model inference, version, 1.43.6., 1, 1–75. https://cran.rproject.org/web/packages/MuMIn/index.html

[eth12986-bib-0005] Beauchamp, G. (2004). Reduced flocking by birds on islands with relaxed predation. Proceedings of the Royal Society B: Biological Sciences, 271(1543), 1039–1042. 10.1098/rspb.2004.2703 PMC1691692 15293857

[eth12986-bib-0006] Beck, K. , Loretto, M.‐C. , & Bugnyar, T. Effects of site fidelity, group size and age on food caching behaviour of common ravens, Corvus corax. Animal Behaviour, submitted.

[eth12986-bib-0007] Belisle, P. , & Chapais, B. (2001). Tolerated co‐feeding in relation to degree of kinship in Japanese macaques. Behaviour, 138(4), 487–509. Retrieved from http://www.jstor.org/stable/4535835

[eth12986-bib-0008] Braun, A. , & Bugnyar, T. (2012). Social bonds and rank acquisition in raven nonbreeder aggregations. Animal Behaviour, 84(6), 1507–1515. 10.1016/j.anbehav.2012.09.024 23264693 PMC3518779

[eth12986-bib-0009] Braun, A. , Walsdorff, T. , Fraser, O. N. , & Bugnyar, T. (2012). Socialized sub‐groups in a temporary stable Raven flock? Journal of Ornithology, 153(Suppl. 1), 97–104. 10.1007/s10336-011-0810-2 25892747 PMC4398859

[eth12986-bib-0010] Bugnyar, T. (2011). Knower–guesser differentiation in ravens: Others' viewpoints matter. Proceedings of the Royal Society B: Biological Sciences, 278(1705), 634–640. 10.1098/rspb.2010.1514 PMC3025684 20826480

[eth12986-bib-0011] Bugnyar, T. , & Heinrich, B. (2005). Ravens, Corvus corax, differentiate between knowledgeable and ignorant competitors. Proceedings of the Royal Society of London. Series B: Biological Sciences, 272(1573), 1641–1646. 16087417 10.1098/rspb.2005.3144 PMC1559847

[eth12986-bib-0012] Bugnyar, T. , & Heinrich, B. (2006). Pilfering ravens, Corvus corax, adjust their behaviour to social context and identity of competitors. Animal Cognition, 9(4), 369–376. 10.1007/s10071-006-0035-6 16909235

[eth12986-bib-0013] Bugnyar, T. , & Kotrschal, K. (2001). Movement coordination and signalling in ravens (corvus corax): An experimental field study. Acta Ethologica, 3(2), 101–109. 10.1007/s102110000029

[eth12986-bib-0014] Bugnyar, T. , & Kotrschal, K. (2002a). Observational learning and the raiding of food caches in ravens, Corvus corax: Is it “tactical” deception? Animal Behaviour, 64(2), 185–195. 10.1006/anbe.2002.3056

[eth12986-bib-0015] Bugnyar, T. , & Kotrschal, K. (2002b). Scrounging tactics in free‐ranging ravens, Corvus corax. Ethology, 108(11), 993–1009. 10.1046/j.1439-0310.2002.00832.x

[eth12986-bib-0016] Bugnyar, T. , & Kotrschal, K. (2004). Leading a conspecffic away from food in ravens (Corvus corax)? Animal Cognition, 7(2), 69–76. 10.1007/s10071-003-0189-4 15069605

[eth12986-bib-0017] Bugnyar, T. , Reber, S. A. , & Buckner, C. (2016). Ravens attribute visual access to unseen competitors. Nature Communications, 7(February), 10506 10.1038/ncomms10506 PMC4740864 26835849

[eth12986-bib-0018] Bugnyar, T. , Schwab, C. , Schloegl, C. , Kotrschal, K. , & Heinrich, B. (2007). Ravens judge competitors through experience with play caching. Current Biology, 17(20), 1804–1808. 10.1016/j.cub.2007.09.048 17949980

[eth12986-bib-0019] Bugnyar, T. , Stöwe, M. , & Heinrich, B. (2007). The ontogeny of caching in ravens, Corvus corax. Animal Behaviour, 74(4), 757–767. 10.1016/j.anbehav.2006.08.019

[eth12986-bib-0020] Burnham, K. P. , & Anderson, D. R. (2002). Model selection and multimodel inference: A practical information‐theoretic approach. New York, NY: Springer.

[eth12986-bib-0021] Byrne, R. W. (1997). The technical intelligence hypothesis: An additional evolutionary stimulus to intelligence? In Machiavellian intelligence II: Extensions and evaluations (pp. 289–311). New York, NY: Cambridge University Press 10.1017/CBO9780511525636.012

[eth12986-bib-0069] Byrne, R. W. , & Whiten, A. (1988a). Tactical deception in primates. Behavioral and Brain Sciences, 11(2), 233–244. 10.1017/s0140525x00049682

[eth12986-bib-0022] Byrne, R. W. , & Whiten, A. (1988b). Machiavellian intelligence: Social expertise and the evolution of intellect in monkeys, apes, and humans. New York, NY: Oxford University Press.

[eth12986-bib-0023] Caffrey, C. (2000). Marking crows. North American Bird Bander, 26(26), 146–148. Retrieved from https://sora.unm.edu/sites/default/files/journals/nabb/v026n04/p0146-p0150.pdf

[eth12986-bib-0024] Clayton, N. S. , Dally, J. M. , & Emery, N. J. (2007). Social cognition by food‐caching corvids. The western scrub‐jay as a natural psychologist. Philosophical Transactions of the Royal Society B: Biological Sciences, 362(1480), 507–522. 10.1098/rstb.2006.1992 PMC2346514 17309867

[eth12986-bib-0025] Coussi‐Korbel, S. (1994). Learning to outwit a competitor in mangabeys (Cercocebus torquatus torquatus). Journal of Comparative Psychology. US: American Psychological Association, 108(2), 164–171. 10.1037/0735-7036.108.2.164 8026168

[eth12986-bib-0026] Dall, S. R. X. , & Wright, J. (2009). Rich pickings near large communal roosts favor “gang” foraging by juvenile common ravens, corvus corax. PLoS ONE, 4(2), e4530 10.1371/journal.pone.0004530 19240813 PMC2646834

[eth12986-bib-0027] Di Bitetti, M. S. , & Janson, C. H. (2001). Social foraging and the finder’s share in capuchin monkeys, Cebus apella. Animal Behaviour, 62(1), 47–56. 10.1006/anbe.2000.1730

[eth12986-bib-0028] Dubuc, C. , & Chapais, B. (2007). Feeding competition in Macaca fascicularis: An assessment of the early arrival tactic. International Journal of Primatology, 28(2), 357–367. 10.1007/s10764-007-9118-8

[eth12986-bib-0029] Dufour, V. , Wascher, C. A. F. , Braun, A. , Miller, R. , & Bugnyar, T. (2012). Corvids can decide if a future exchange is worth waiting for. Biology Letters, 8(2), 201–204. Retrieved from http://rsbl.royalsocietypublishing.org/content/8/2/201.abstract. 10.1098/rsbl.2011.0726 21920957 PMC3297375

[eth12986-bib-0030] Dunbar, R. I. M. (2003). The social brain: Mind, language, and society in evolutionary perspective. Annual Review of Anthropology, 32(1), 163–181. 10.1146/annurev.anthro.32.061002.093158

[eth12986-bib-0031] Emery, N. J. , & Clayton, N. S. (2001). Effects of experience and social context on prospective caching strategies by scrub jays. Nature, 414(6862), 443–446. 10.1038/35106560 11719804

[eth12986-bib-0032] Flower, T. P. , Gribble, M. , & Ridley, A. R. (2014). Deception by flexible alarm mimicry in an African bird. Science, 344(6183), 513–516. 10.1126/science.1249723 24786078

[eth12986-bib-0033] Fournier, D. A. , Skaug, H. J. , Ancheta, J. , Ianelli, J. , Magnusson, A. , Maunder, M. N. , … Sibert, J. (2012). AD Model Builder: Using automatic differentiation for statistical inference of highly parameterized complex nonlinear models. Optimization Methods and Software, 27(2), 233–249. 10.1080/10556788.2011.597854

[eth12986-bib-0034] Fox, J. , & Weisberg, S. (2011). An R companion to applied regression (Second). Thousand Oaks, CA: Sage Retrieved from http://socserv.socsci.mcmaster.ca/jfox/Books/Companion

[eth12986-bib-0035] Fraser, O. N. , Schino, G. , & Aureli, F. (2008). Components of relationship quality in chimpanzees. Ethology, 114(9), 834–843. 10.1111/j.1439-0310.2008.01527.x

[eth12986-bib-0036] Giraldeau, L. A. , Caraco, T. (2000). Social foraging theory. Ecology (vol. 82). Princeton, NJ: Princeton University Press 10.2307/2680211

[eth12986-bib-0037] Goss‐Custard, J. D. , & Durell, S. (1988). The effect of dominance and feeding method on the intake rates of oystercatchers, Haematopus ostralegus, feeding on mussels. The Journal of Animal Ecology, 57(3), 827 10.2307/5095

[eth12986-bib-0038] Grand, T. C. , & Dill, L. M. (1999). The effect of group size on the foraging behaviour of juvenile Coho salmon: Reduction of predation risk or increased competition? Animal Behaviour, 58(2), 443–451. 10.1006/anbe.1999.1174 10458896

[eth12986-bib-0039] Gwinner, E. (1965). U¨ ber den Einfluß des Hungers und andere Faktoren auf die Versteck-Aktivita¨ t des Kolkraben (Corvus corax). Vogelwarte, 23, 1–4.

[eth12986-bib-0040] Heinrich, B. (1988). Winter foraging at carcasses by three sympatric corvids, with emphasis on recruitment by the raven, Corvus corax. Behavioral Ecology and Sociobiology, 23(3), 141–156. 10.1007/BF00300349

[eth12986-bib-0041] Heinrich, B. (1989). Ravens in winter. New York, NY: Vintage Books Retrieved from https://books.google.at/books?xml:id=YbWtadkEVvUC

[eth12986-bib-0042] Heinrich, B. , Kaye, D. , Knight, T. , & Schaumburg, K. (1994). Dispersal and association among common ravens. The Condor, 96(2), 545–551. 10.2307/1369334

[eth12986-bib-0043] Heinrich, B. , & Marzluff, J. M. (1991). Do common ravens yell because they want to attract others? Behavioral Ecology and Sociobiology, 28(1), 13–21. 10.1007/BF00172134

[eth12986-bib-0044] Heinrich, B. , & Marzluff, J. (1992). Age and mouth color in common ravens. The Condor, 94(2), 549–550. 10.1242/jeb.089763

[eth12986-bib-0045] Heinrich, B. , & Pepper, J. W. (1998). Influence of competitors on caching behaviour in the common raven, Corvus corax. Animal Behaviour, 56, 1083–1090. 10.1006/anbe.1998.0906 9819322

[eth12986-bib-0046] Held, S. , Mendl, M. , Devereux, C. , & Byrne, R. W. (2002). Foraging pigs alter their behaviour in response to exploitation. Animal Behaviour, 64(2), 157–165. 10.1006/anbe.2002.3044

[eth12986-bib-0047] Hillemann, F. , Bugnyar, T. , Kotrschal, K. , & Wascher, C. A. F. (2014). Waiting for better, not for more: Corvids respond to quality in two delay maintenance tasks. Animal Behaviour, 90, 1–10. 10.1016/j.anbehav.2014.01.007 25892738 PMC4398876

[eth12986-bib-0048] Hirata, S. , & Matsuzawa, T. (2001). Tactics to obtain a hidden food item in chimpanzee pairs (Pan troglodytes). Animal Cognition, 4(3), 285–295. 10.1007/s100710100096 24777519

[eth12986-bib-0049] Humphrey, N. (1976). The social function of intellect In Bateson P. P. G. & Hinde R. A. (Eds.), Growing Points in Ethology (pp. 303–317). Cambridge, UK: Cambridge University Press.

[eth12986-bib-0050] Jolly, A. (1966). Lemur social behavior and primate intelligence. Science, 153(3735), 501–506. 10.1126/science.153.3735.501 5938775

[eth12986-bib-0051] Loretto, M.‐C. , Schuster, R. , & Bugnyar, T. (2016). GPS tracking of non‐breeding ravens reveals the importance of anthropogenic food sources during their dispersal in the Eastern Alps. Current Zoology, 62(4), 337–344. 10.1093/cz/zow016 29491922 PMC5829441

[eth12986-bib-0052] Loretto, M.‐C. , Schuster, R. , Itty, C. , Marchand, P. , Genero, F. , & Bugnyar, T. (2017). Fission‐fusion dynamics over large distances in raven non‐breeders. Scientific Reports, 7(1), 380 10.1038/s41598-017-00404-4 28336913 PMC5428508

[eth12986-bib-0053] Marzluff, J. M. , & Heinrich, B. (1991). Foraging by common ravens in the presence and absence of territory holders: An experimental analysis of social foraging. Animal Behaviour, 42(5), 755–770. 10.1016/S0003-3472(05)80121-6

[eth12986-bib-0054] Marzluff, J. M. , Heinrich, B. , & Marzluff, C. S. (1996). Raven roosts are mobile information centres. Animal Behaviour, 51(1), 89–103. 10.1006/anbe.2000.1579

[eth12986-bib-0055] Menzel, E. W. (1974). Chapter 3 ‐ a group of young chimpanzees in a one‐acre field: Leadership and communication In Schrier A. M., & Stollnitz F. (Eds.), Behaviour of nonhuman primates, Vol. 5 (pp. 83–153). San Diego, CA: Academic Press 10.1016/B978-0-12-629105-6.50009-2

[eth12986-bib-0056] Morand‐Ferron, J. , Sol, D. , & Lefebvre, L. (2007). Food stealing in birds: Brain or brawn? Animal Behaviour, 74(6), 1725–1734. 10.1016/j.anbehav.2007.04.031

[eth12986-bib-0057] Munn, C. A. (1986). Birds that ‘cry wolf’. Nature, 319(6049), 143–145. 10.1038/319143a0

[eth12986-bib-0058] Nácarová, J. , Veselý, P. , & Bugnyar, T. (2018). Ravens adjust their antipredatory responses to con‐ ­ and specific alarms to the perceived threat. Ethology, 124, 1–8. 10.1111/eth.12764

[eth12986-bib-0059] Parker, S. T. , & Gibson, K. R. (1977). Object manipulation, tool use and sensorimotor intelligence as feeding adaptations in cebus monkeys and great apes. Journal of Human Evolution, 6(7), 623–641. 10.1016/S0047-2484(77)80135-8

[eth12986-bib-0060] Powell, G. V. N. (1974). Experimental analysis of the social value of flocking by starlings (Sturnus vulgaris) in relation to predation and foraging. Animal Behaviour, 22(2), 501–505. 10.1016/S0003-3472(74)80049-7

[eth12986-bib-0061] R Core Development Team (2014). R: a language and environment for statistical computing. Vienna, Austria: R Foundation for Statistical Computing.

[eth12986-bib-0062] Rosati, A. G. (2017). Foraging cognition: Reviving the ecological intelligence hypothesis. Trends in Cognitive Sciences, 21(9), 691–702. 10.1016/j.tics.2017.05.011 28625354

[eth12986-bib-0063] Symonds, M. R. E. , & Moussalli, A. (2011). A brief guide to model selection, multimodel inference and model averaging in behavioural ecology using Akaike’s information criterion. Behavioral Ecology and Sociobiology, 65(1), 13–21. 10.1007/s00265-010-1037-6

[eth12986-bib-0064] Szipl, G. , Ringler, E. , Spreafico, M. , & Bugnyar, T. (2017). Calls during agonistic interactions vary with arousal and raise audience attention in ravens. Frontiers in Zoology, 14(1), 1–13. 10.1186/s12983-017-0244-7 29299036 PMC5740903

[eth12986-bib-0065] Templeton, J. J. , & Giraldeau, L. A. (1995). Patch assessment in foraging flocks of european starlings: Evidence for the use of public information. Behavioral Ecology, 6(1), 65–72. 10.1093/beheco/6.1.65

[eth12986-bib-0066] Templeton, J. J. , & Giraldeau, L. A. (1996). Vicarious sampling: The use of personal and public information by starlings foraging in a simple patchy environment. Behavioral Ecology and Sociobiology, 38, 105–114. 10.1007/s002650050223

[eth12986-bib-0067] Vickery, W. L. , Giraldeau, L. ‐A. , Templeton, J. J. , Kramer, D. L. , & Chapman, C. A. (1991). Producers, scroungers and group foraging. The American naturalist, 137(6), 847–863.

[eth12986-bib-0068] Webb, W. C. (2004). Common raven juvenile survival in a human‐augmented landscape, (February 2014). 10.1650/7443

[eth12986-bib-0070] Wright, J. , Stone, R. E. , & Brown, N. (2003). Communal roost as structured information centre in the rave, Corvus corax. Journal of Animal Ecology, 72(6), 1003–1014. 10.1046/j.1365-2656.2003.00771.x

[eth12986-bib-0071] Wunderle, J. M. J. (1991). Age‐specific foraging proficiency in birds. Current Ornithology, 8, 273–324. Retrieved from 10017456741/en/

