# Supplementary material for: Decision time modulates social foraging success in wild common ravens, Corvus corax
Source: Ethology. 2019 Nov 24;126(4):413–22. doi: 10.1111/eth.12986 (PMC7079088; doi:10.1111/eth.12986)
Supplement: Supplementary file 1 [file ETH-126-413-s001.docx]

**Supplementary material.** Gallego-Abenza, M. Loretto, M-C. & Bugnyar, T. Decision time modulates foraging success in wild common ravens, *Corvus corax*

**Table S1** showing the estimates of the full model explaining **general foraging success** before model averaging.

|  | **Estimate ± SE** | **Z value** | **P** |
| --- | --- | --- | --- |
| *Intercept* | 1.146 ± 0.301 | 3.81 | 0.00014 |
| Age class (Subadult) | -0.915 ± 0.367 | -2.50 | 0.01257 |
| Age class (Juvenile) | -2.392 ± 0.504 | -4.74 | 2.1 x 10^-6^ |
| Distance to conspecific | 0.160 ± 0.105 | 1.53 | 0.12699 |
| Decision time | 0.313 ± 0.127 | 2.48 | 0.01326 |
| Number of surrounding ravens | -0.142 ± 0.101 | -1.41 | 0.16002 |
| Sex (male) | 0.471 ± 0.354 | 1.33 | 0.18357 |
| Percentage of days being present | 0.144 ± 0.123 | 1.17 | 0.24287 |
| Winning probability | -0.142 ± 0.183 | -0.78 | 0.43733 |

**Table S2** showing the estimates of the full model explaining **consuming food directly on site success** before model averaging.

|  | **Estimate ± SE** | **Z value** | **P** |
| --- | --- | --- | --- |
| *Intercept* | -3.406 ± 1.525 | -2.23 | 0.025 |
| Age class (Subadult) | -0.146 ± 1.406 | -0.10 | 0.9171 |
| Age class (Juvenile) | -0.753 ± 1.691 | -0.45 | 0.6562 |
| Distance to conspecific | 0.363 ± 0.291 | 1.25 | 0.2124 |
| Decision time | 0.975 ± 0311 | 3.14 | 0.0017 |
| Number of surrounding ravens | 0.128 ± 0.355 | 0.36 | 0.7195 |
| Sex (male) | 1.840 ± 1.154 | 1.59 | 0.1108 |
| Percentage of days being present | 0.125 ± 0.421 | 0.30 | 0.7659 |
| Winning probability | 0.614 ± 0.674 | 0.91 | 0.3620 |

**Table S3** showing the estimates of the full model explaining **carrying food away decision** before model averaging.

|  | **Estimate ± SE** | **Z value** | **P** |
| --- | --- | --- | --- |
| *Intercept* | 2.3017 ± 0.3700 | 6.22 | 5 x 10^-10^ |
| Age class (Subadult) | -0.7980 ± 0.4366 | -1.83 | 0.068 |
| Age class (Juvenile) | -2.2873 ± 0.5379 | -4.25 | 2.1 x 10^-5^ |
| Distance to conspecific | -0.0524 ± 0.1071 | -0.49 | 0.625 |
| Decision time | 0.0638 ± 0.0895 | 0.71 | 0.476 |
| Number of surrounding ravens | 0.2970 ± 0.1167 | 2.55 | 0.011 |
| Sex (male) | -0.3802 ± 0.3943 | -0.96 | 0.335 |
| Percentage of days being present | -0.0142 ± 0.1399 | -0.10 | 0.919 |
| Winning probability | 0.0945 ± 0.2043 | 0.46 | 0.644 |

**Table S4** showing the estimates of original model explaining **carrying food away success** before model averaging.

|  | **Estimate ± SE** | **Z value** | **P** |
| --- | --- | --- | --- |
| *Intercept* | 1.632 ± 0.227 | 7.18 | 6.9 x 10^-13^ |
| Age class (Subadult) | -0.776 ± 0.311 | -2.49 | 0.0126 |
| Age class (Juvenile) | -1.504 ± 0.477 | -3.15 | 0.0016 |
| Distance to conspecific | 0.313 ± 0.151 | 2.07 | 0.0381 |
| Decision time | 0.186 ± 0.186 | 0.159 | 0.2426 |
| Number of surrounding ravens | -0.342 ± 0.126 | -2.71 | 0.0067 |
| Sex (male) | 0.575 ± 0.277 | 2.08 | 0.0377 |
| Percentage of days being present | 0.144 ± 0.133 | 1.08 | 0.2810 |
| Winning probability | -0.195 ± 0.157 | -1.24 | 0.2146 |

**Table S5** showing the percentage of kleptoparasitized food saving attempts, together with the age class and sex information for each individual.

| **Individual** | **Age class** | **Sex** | **Winning probability** | **Percentage of kleptoparasitized food retention attempts** |
| --- | --- | --- | --- | --- |
| Aletheia | Juvenile | female | 0.10 | 77.78 |
| Arya | Subadult | male | 0.75 | 48.89 |
| Athena | Subadult | female | 0.25 | 0.00 |
| Baal | Subadult | male | 0.60 | 75.00 |
| Basil | Juvenile | female | 0.33 | 100.00 |
| Cheyenne | Subadult | male | 0.31 | 30.77 |
| Chili | Subadult | female | 0.25 | 87.50 |
| Clove | Juvenile | male | 0.41 | 25.00 |
| Eva-Maria | Juvenile | male | 0.00 | 0.00 |
| Ford | Adult | male | 1.00 | 21.43 |
| Fuchur | Adult | female | 0.53 | 35.71 |
| Gendarmer | Juvenile | male | 0.00 | 100.00 |
| Gigolo | Adult | female | 0.00 | 16.67 |
| Gisla | Subadult | female | 0.35 | 63.64 |
| Hedda | Adult | female | 0.50 | 41.18 |
| Hektor | Adult | female | 0.00 | 28.57 |
| Helsinki | Adult | female | 0.67 | 31.25 |
| Hierro | Juvenile | male | 0.19 | 42.86 |
| Hillary | Adult | female | 0.25 | 28.57 |
| Hydra | Subadult | female | 0.17 | 100.00 |
| Ivy | Juvenile | female | 0.22 | 50.00 |
| Johnny | Juvenile | female | 0.00 | 100.00 |
| Josefa | Adult | male | 0.50 | 21.74 |
| Kalima | Subadult | female | 0.06 | 50.00 |
| Kassiopeia | Adult | female | 0.80 | 21.74 |
| Keiwu | Juvenile | female | 0.16 | 48.15 |
| Kurt | Juvenile | male | 0.16 | 80.00 |
| Lydia | Juvenile | male | 0.55 | 66.67 |
| Maila | Adult | female | 0.86 | 17.89 |
| Mara | Subadult | female | 0.30 | 62.79 |
| Mario | Juvenile | male | 0.03 | 100.00 |
| Merlin | Juvenile | male | 1.00 | 100.00 |
| Parzival | Juvenile | female | 0.00 | 100.00 |
| Pueblo | Subadult | male | 0.90 | 66.67 |
| Regulus | Subadult | male | 0.93 | 34.29 |
| Richy | Juvenile | male | 0.20 | 100.00 |
| Rollo | Subadult | male | 0.31 | 22.22 |
| Rory | Subadult | female | 0.00 | 0.00 |
| Salt | Subadult | male | 0.53 | 28.57 |
| Sichuan | Juvenile | male | 0.00 | 12.50 |
| Sixto | Adult | female | 0.42 | 35.71 |
| Solitary | Subadult | female | 0.38 | 29.03 |
| Source | Juvenile | female | 0.00 | 100.00 |
| Timon | Subadult | male | 0.63 | 83.33 |
| Voldemort | Adult | male | 1.00 | 0.00 |
| Yakuza | Subadult | female | 0.55 | 45.05 |
